# Antiosteoporosis medications and cardiovascular disease: a population-based nationwide nested case–control study

**DOI:** 10.3389/fphar.2023.1220174

**Published:** 2023-10-10

**Authors:** Wen-Hsuan Tsai, Fung-Chang Sung, Chih-Hsin Muo, Ming-Chieh Tsai, Shu-I. Wu

**Affiliations:** ^1^ Division of Endocrinology and Metabolism, Department of Internal Medicine, Mackay Memorial Hospital, Taipei, Taiwan; ^2^ Management Office for Health Data, Clinical Trial Research Center, China Medical University Hospital, Taichung, Taiwan; ^3^ Department of Health Services Administration, China Medical University College of Public Health, Taichung, Taiwan; ^4^ Department of Food Nutrition and Health Biotechnology, Asia University, Taichung, Taiwan; ^5^ Department of Psychiatry, Mackay Memorial Hospital, Taipei, Taiwan; ^6^ Department of Medicine, MacKay Medical College, New Taipei City, Taiwan

**Keywords:** bisphosphonate, denosumab, teriparatide, hormone replacement therapy, cardiovascular disease

## Abstract

**Purpose:** Patients with osteoporosis are at an increased risk of cardiovascular disease (CVD). Several antiosteoporosis medications have been demonstrated with the benefit of preventing osteoporosis. Our aim is to assess the CVD risks associated with antiosteoporosis medications using the National Health Insurance Research Database in Taiwan between 2000 and 2016.

**Methods:** Among 41,102 patients of 40+ years old with newly diagnosed osteoporosis, 69.1% (N = 28,387) of patients were included in the user cohort of antiosteoporosis medicines, of whom 13, 472 developed CVD by the end of 2016, while 14,915 did not. Using the nested case–control analysis in the user cohort (88.0% women and 77.4% elderly), we applied conditional logistic regression to estimate odds ratios (ORs) of eight types of CVD for the users of denosumab, bisphosphonate, teriparatide, and hormone replacement therapy (HRT).

**Results:** The adjusted ORs of overall CVDs were 0.13 (95% CI: 0.12–0.15) for denosumab users, 0.52 (95% CI: 0.45–0.61) for teriparatide users, and 0.80 (95% CI: 0.76–0.85) for bisphosphonate users. The HRT users were at higher odds of coronary artery and peripheral artery diseases, heart failure, pulmonary embolism, and deep vein thrombosis.

**Conclusion:** Denosumab, teriparatide, and bisphosphonate may have more protective effects against CVD than hormone therapy. Physicians may take subsequent cardiovascular risks into account when choosing an adequate antiosteoporosis medication for patients with osteoporosis.

## Introduction

Both osteoporosis and cardiovascular diseases (CVDs) are disorders associated with aging, and they may share some similar mechanisms (Fuggle et al., 2020). Atherosclerosis and osteoporosis may be associated with the net flux of calcium shifting from skeleton to depositions in the kidney, vessel walls, and other soft tissues (Nicoll and Henein, 2017). The mechanisms underlying aging, such as chronic inflammation or decline in renal function that affects the calcium level and mineral balance, are thought to interact with the eventual co-manifestations of osteoporosis and CVD (Rodríguez and Abrahamsen, 2021). The association between bone and vascular diseases may be bi-directional (Rodriguez et al., 2019). For instance, previous studies have shown an increased prevalence of coronary artery diseases (CADs) and cardiovascular events among individuals with osteoporosis (Marcovitz et al., 2005; Tankó et al., 2005; Varma et al., 2008). Patients with osteoporosis were also found to have a two-fold increased risk of cardiovascular mortality (von der Recke et al., 1999).

Antiosteoporosis medications recommended for osteoporosis treatment have been associated with the development of CVD with conflicting findings. Although an antiosteoporosis medication, romosozumab, has more protective effects in preventing fracture than alendronate, incident cardiac ischemic events are increased (Saag et al., 2017). Lyles et al. showed an 11% reduction of risks in cardiovascular events and a 31% reduction of cardiovascular deaths, after the use of bisphosphonates (Lyles et al., 2007). Cohort studies also showed that bisphosphonates were associated with a decreased risk of myocardial infarction (Kang et al., 2012; Vestergaard, 2012; Kang et al., 2013; Wolfe et al., 2013). On the other hand, a meta-analysis indicated a higher risk of CVD comparing denosumab to bisphosphonates in postmenopausal women (Seeto et al., 2021). Findings from other meta-analyses indicated that this might be because denosumab has no impact on cardiovascular risks (Ferrieres et al., 2020; Lv et al., 2020). A previous randomized controlled trial (RCT) showed a neutral effect of abaloparatide and teriparatide on major adverse cardiovascular events (MACEs) when compared with placebo (Cosman et al., 2020). When adding heart failure (HF) to MACEs, abaloparatide and teriparatide had a significantly lower risk of MACE plus HF when compared with the placebo (Cosman et al., 2020). However, when abaloparatide was shifted to alendronate, the protective effect became neutral in the extended phase (Cosman et al., 2020). A meta-analysis demonstrated that parathyroid hormone (PTH) analogs have no impact on cardiovascular risks and overall mortality (Ferrieres et al., 2020).

Postmenopausal hormone replacement therapy (HRT), a well-known osteoporotic fracture prevention choice for postmenopausal women, has been associated with a two-fold increased risk for venous thromboembolism (VTE) (Miller et al., 2002). Studies using head-to-head comparisons of HRT, bisphosphonate, denosumab, and teriparatide on the risks of different CVDs are still lacking. Hence, we used a large, population-based database comprising insurance claims in Taiwan to conduct a retrospective cohort study comparing CVD events that had occurred between the antiosteoporosis medication users (user cohort) and those without the medications (non-user cohort). We further used a nested case–control analysis within the user cohort to assess incident CVDs associated with the uses of different antiosteoporosis medications.

## Methods and materials

### Data source

The Taiwan National Health Insurance (NHI) program is a compulsory health insurance system launched in 1995 for all residents in Taiwan. For this study, we used the National Health Insurance Research Database (NHIRD) of 2000–2016 containing claims’ data on all insured individuals. The claims data provided records of birth date, sex, income, and occupation, as well as diagnoses, drug prescriptions, and treatments from emergency, outpatient visits, and hospitalizations from 2000 to 2016. All included enrollees in the NHIRD had de-identified numbers and were analyzed anonymously; hence, consents were waived. This study was approved by the Institutional Review Board of Mackay Memorial Hospital (22MMHIS140e).

### Study design

#### Establishing study cohorts and follow-up outcomes

Antiosteoporosis medications were available treatment options for patients with osteoporosis. We first identified 10,760,702 residents older or equal to 40 years of age covered by the insurance during the period of 2000–2016 (Supplementary Figure S1). We excluded patients with a history of CVD, those without osteoporosis, those with osteoporosis but visited the clinic less than three times, or those who used antiosteoporosis medication prior to the diagnosis of osteoporosis. Patients who were newly diagnosed with osteoporosis (ICD-10 codes: M81.0, M81.1, M81.2, M81.5, M81.6, M81.8, and M81.9 and ICD-9-CM codes: 733.00, 733.01, 733.02, and 733.09) at least three times from the outpatient clinic between 2000 and 2016 were considered the potential study population. The date of the initial diagnosis with osteoporosis was designated as the index date.

Patients diagnosed with osteoporosis were divided into the user and non-user cohorts, with and without using antiosteoporosis medications, including denosumab, teriparatide, bisphosphonate, and/or HRT (Supplementary Figure S1). Patients in both cohorts were followed until incident CVDs were identified, withdrawn from the insurance, or the end of 2016. Our study aimed to evaluate eight types of CVD, namely, coronary artery diseases (CADs), CVD, HF, peripheral artery disease (PAD), atrial fibrillation (Af), arrhythmia other than Af, pulmonary embolism (PE), and deep vein thrombosis (DVT) (detailed ICD-9-CM and ICD-10 diagnoses may be found in our Supplementary Table S1). The incidence rate of each CVD was estimated per 1,000 person-years.

#### Covariates

Covariates that might be associated with developing CVDs were included in the analyses. In addition to demographic characteristics, we considered baseline comorbidities of diabetes (DM), hyperlipidemia, hypertension, chronic kidney disease (CKD), chronic obstructive pulmonary disease (COPD), obesity, malignancy, liver cirrhosis, end-stage renal disease (ESRD), and osteoporotic fracture. The detailed ICD-9-CM or ICD-10 diagnoses may be found in Supplementary Table S1.

#### Nested case–control analysis

At the end of a follow-up, with the incident CVD events identified, we further conducted a case–control analysis within the user cohort to evaluate factors associated with the CVD events. Patients who had been prescribed medications of denosumab, bisphosphonate, teriparatide, and HRT within 1 year before the endpoint of follow-up were evaluated for the CVD events.

### Statistical analysis

Distributions of sex, age, income, occupation, and comorbidities were compared between the user and non-user cohorts of antiosteoporosis medications. A chi-squared test was used to examine categorical variables and t-tests for continuous variables. We estimated the lifetime incidence rates of each type of CVD for both cohorts. Cox hazard regression analysis was used to estimate the user cohort to non-user cohort crude hazard ratio (cHR) of each type of CVD and related 95% confidence interval (CI). The adjusted hazard ratio (aHR) was estimated after controlling for covariates. In the case–control analysis, we first compared distributions of CVD cases and non-cases by demographic factors, comorbidities, ever fracture, and antiosteoporosis medication used within 1 year before the endpoint (within 1 year by the time with CVD diagnosed or 1 year before withdrawal from the insurance or the end of the follow-up), including denosumab, bisphosphonate, teriparatide, and HRT. Conditional logistic regression was applied to calculate OR and 95% CI of overall CVD. Adjustments were made after controlling for the aforementioned covariates. We further calculated the OR for each type of CVD associated with each antiosteoporosis medication within 1 year before the endpoint. All statistical analyses were performed using STATA version 14.0 (StataCorp), and results with *p*-values less than 0.05 were considered statistically significant.

## Results

Among the 41,102 patients aged ≥40 years with newly diagnosed osteoporosis from 2000 to 2016, 69.1% of patients (N = 28,387) were included in the user cohort and 30.9% (N = 12,715) were included in the non-user cohort (Supplementary Figure S1 and Table 1). The user cohort had a higher proportion of women, younger patients, higher income, fewer comorbidities, and fewer events of osteoporotic fracture than non-users (Table 1).

**TABLE 1:** Baseline characteristics of study cohorts of users and non-users of antiosteoporosis medications.

	Antiosteoporosis medications						
	Yes N = 28,387		No N = 12,715		Total N = 41,102		
Characteristic	n	%	n	%	n	%	*p*-value
**Sex**							<0.0001
Men	3,399	12.0	3,455	27.2	6,854	16.7	
Women	24,988	88.0	9,260	72.8	34,248	83.3	
**Age**							<0.0001
40–49	526	1.85	301	2.37	827	2.01	
50–64	5,883	20.7	2,333	18.4	8,216	20.0	
65+	21,978	77.4	10,081	79.3	32,059	78.0	
**Income, NTD**							<0.0001
<19,200	66,120	23.3	3,482	27.4	10,102	24.6	
19,200–1,999	15,003	52.9	6,381	50.2	21,384	52.0	
20,000+	6,764	23.8	2,852	22.4	9,616	23.4	
**Occupation**							<0.0001
Housekeeping	9,915	34.9	4,052	31.9	13,967	34.0	
White collar	2,087	7.35	1,020	8.02	3,107	7.56	
Blue collar	13,594	47.9	5,638	44.3	19,232	46.8	
Other	2,791	9.83	2,005	15.8	4,796	11.7	
**Comorbidity**							
Diabetes mellitus	5,155	18.2	2,604	20.5	7,759	18.9	<0.0001
Hyperlipidemia	4,098	14.4	1,886	14.8	5,984	14.6	0.292
Hypertension	12,368	46.6	5,997	47.2	18,365	44.7	<0.0001
CKD	3,505	12.4	1,804	14.2	5,309	12.9	<0.0001
COPD	6,938	24.4	3,396	26.7	10,334	25.1	<0.0001
Obesity	85	0.30	39	0.31	124	0.30	0.901
ESRD	2,444	8.61	1,332	10.5	3,776	9.19	<0.0001
Malignancy	2,034	7.17	971	7.64	3,005	7.31	0.090
Cirrhosis	4,272	15.1	1,958	15.4	6,230	15.2	0.360
Osteoporosis fracture	8,080	28.5	4,237	33.3	12,317	30.0	<0.0001

NTD, New Taiwan dollar; CKD, chronic kidney disease; COPD, chronic obstructive pulmonary disease; ESRD, end-stage renal disease.

Table 2 shows that the user cohort had a significantly lower aHR value of 0.93 (95% CI, 0.89–0.97) for heart failure but had higher aHR values of 1.15 (95% CI 1.00–1.33) for DVT and 1.12 (95% CI 1.05–1.19) for arrhythmia other than Af. The HRs of CAD, CVD, PAD, Af, and PE were not significantly different between the two cohorts.

**TABLE 2:** Lifetime incidence and hazard ratios of cardiovascular disease associated with antiosteoporosis medications.

	Antiosteoporosis medications							
	Yes N = 28,387			No N = 12,715			Hazard ratio (95% confidence interval)	

Cardiovascular disease	n	PY	Rate	n	PY	Rate	Crude	Adjusted
Coronary artery disease	6,771	256,102	26.4	2,586	93,803	27.6	0.99 (0.94–1.03)	1.04 (0.99–1.09)
Cerebrovascular disease	115	301,765	0.38	30	107,741	0.28	1.04 (0.69–1.55)	1.24 (0.82–1.88)
Peripheral artery disease	3,358	280,131	12.0	1,331	100,619	13.2	0.92 (0.86–0.98)	0.97 (0.90–1.03)
Heart failure	8,426	253,769	33.2	3,504	92,127	38.0	0.88 (0.85–0.92)	0.93 (0.89–0.97)
Atrial fibrillation	2,589	291,945	8.87	1,064	104,194	10.2	0.84 (0.78–0.90)	0.95 (0.88–1.02)
Arrhythmia other than atrial fibrillation	4,176	272,230	15.3	1,428	99,832	14.3	1.09 (1.03–1.16)	1.12 (1.05–1.19)
Pulmonary embolism	324	300,664	1.08	96	107,479	0.89	1.16 (0.92–1.46)	1.18 (0.93–1.48)
Deep vein thrombosis	831	297,659	2.79	260	106,574	2.44	1.13 (0.98–1.30)	1.15 (1.00–1.33)

PY, person years; adjusted hazard ratio: estimated after controlling for age, sex, income, occupation, diabetes mellitus, hypertension, chronic kidney disease, chronic obstructive pulmonary disease, end-stage renal disease, osteoporosis fracture, and medicine.

**FIGURE 1:**
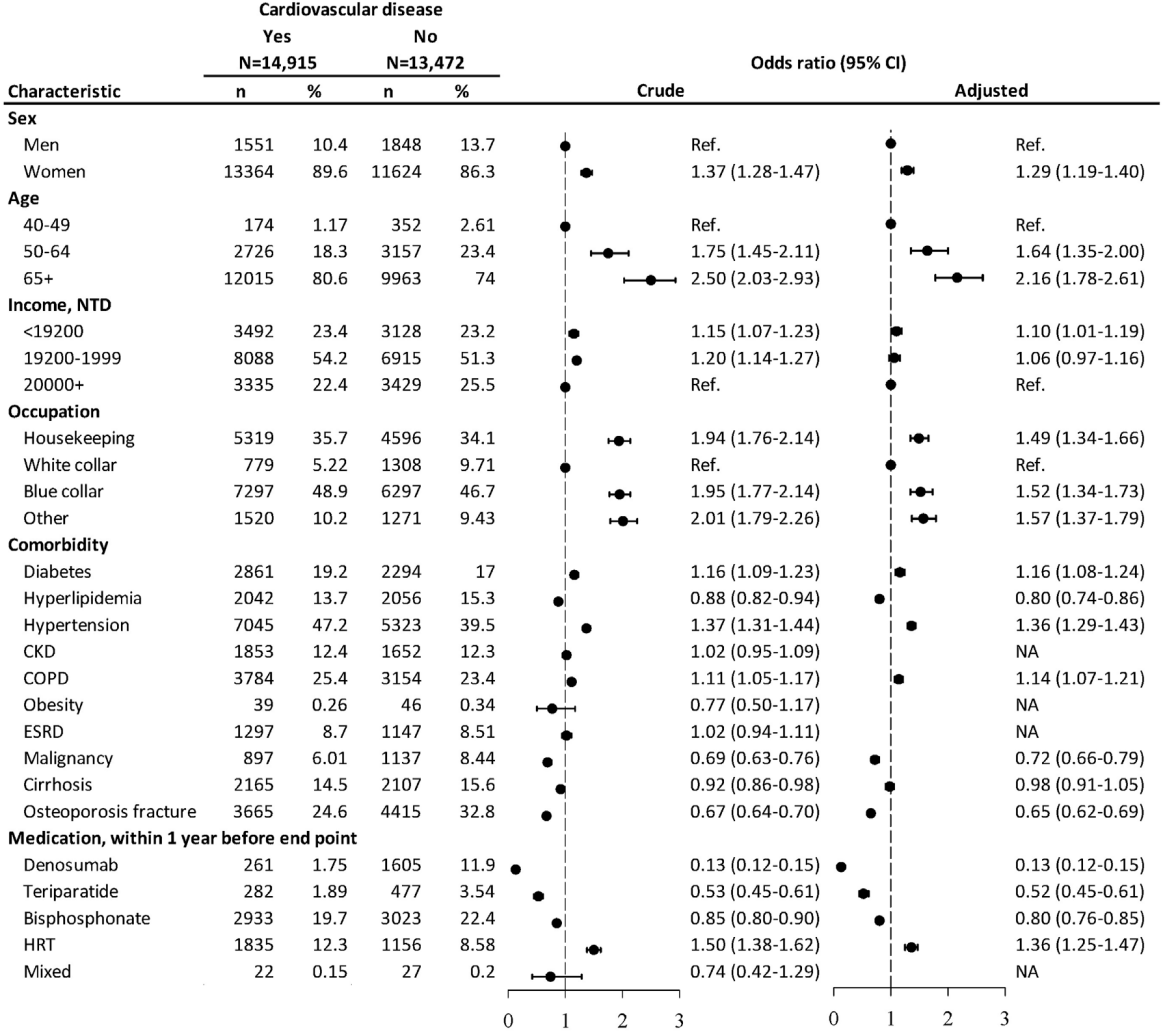
Nested case–control analysis within the antiosteoporosis medication cohort assessing factors associated with cardiovascular diseases. NTD, New Taiwan dollar; CKD, chronic kidney disease; COPD, chronic obstructive pulmonary disease; ESRD, end-stage renal disease. Adjusted odds ratio: estimated after controlling for age, sex, income, occupation, diabetes mellitus, hyperlipidemia, hypertension, COPD, malignancy, cirrhosis, osteoporosis fracture, and medication.

From the nested case-controlled analysis, evaluating factors associating with CVD, women, aged 50 and above, with the least income, non-white collar workers, those with DM, hypertension, or COPD were associated with increased risks of CVD (Figure 1). Patients with hyperlipidemia, malignancy, or osteoporosis fracture were associated with decreased risks of CVD. The CVD risk was lowered in patients using denosumab (OR = 0.13, 95% CI: 0.12–0.15), teriparatide (OR = 0.52, 95% CI: 0.45–0.61), and bisphosphonate (OR = 0.80, 95% CI: 0.76–0.85) within 1 year before the endpoint. The user of HRT was associated with higher odds of CVD (OR = 1.36, 95% CI: 1.25–1.47).

**FIGURE 2:**
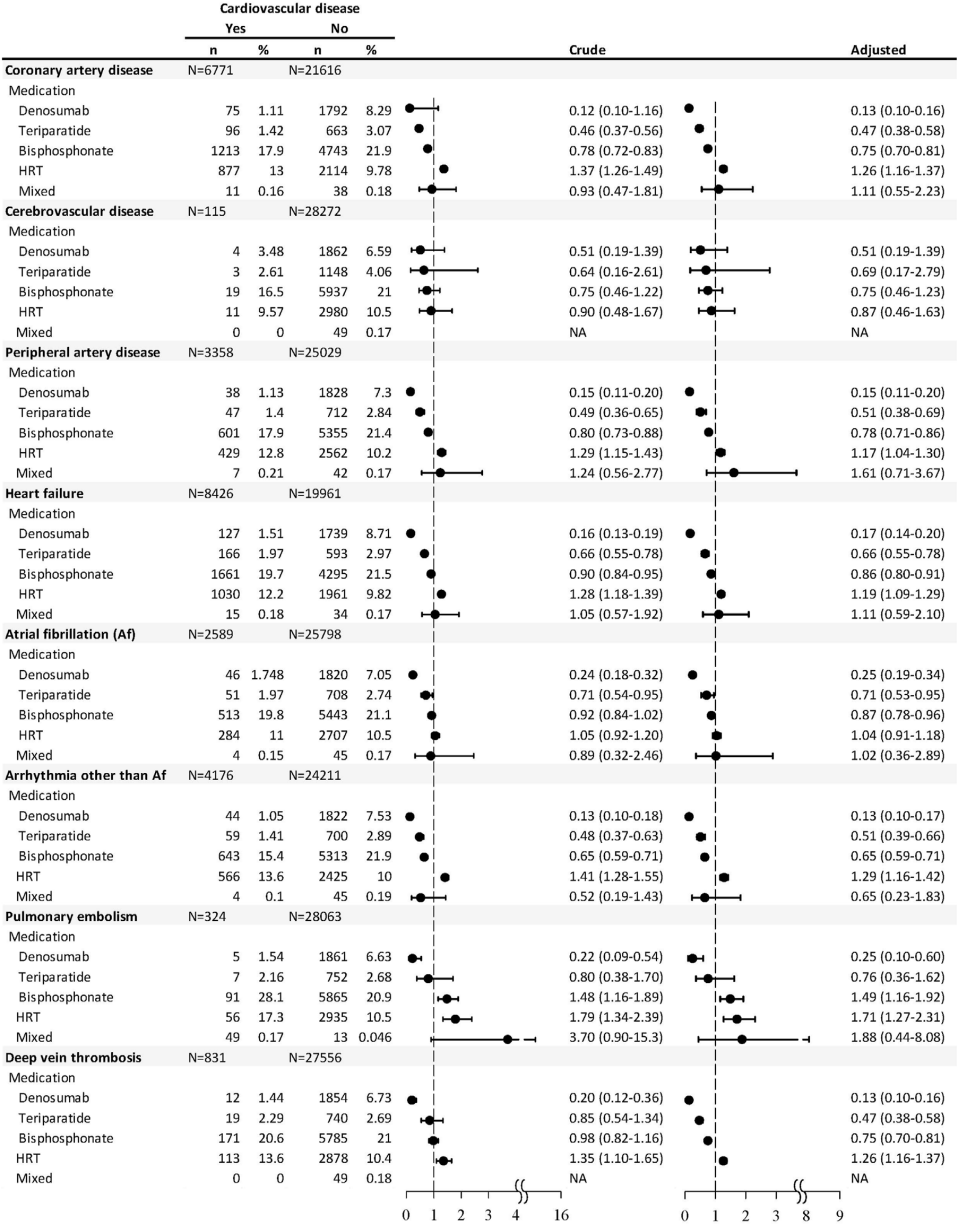
Nested case–control analysis within the antiosteoporosis medication cohort estimating the odds ratio of each type of cardiovascular disease associated with antiosteoporosis medication used within 1 year before the endpoint.

As for each CVD, users of denosumab, teriparatide, and bisphosphonate, within 1 year before the end point, were significantly associated with lower odds of CAD, PAD, HF, arrhythmia other than Af, and DVT, while the users of HRT were associated with higher odds of these cardiovascular events (Figure 2). Users of denosumab or teriparatide were associated with lower odds of Af, while users of bisphosphonate or HRT were associated with higher odds of PE. Patients with HRT use were associated with higher odds of DVT (OR 1.26, 95% CI 1.16–1.37), but such risks were significantly lower among the users of denosumab (OR 0.13, 95% CI 0.10–0.16), teriparatide (OR 0.47, 95% CI 0.38–0.58), or bisphosphonate (OR 0.75, 95% CI 0.70–0.81). There was no significant association between CVD and medications used in the last year of follow-up.

## Discussion

This is the first population-based study evaluating the CVD risk among patients using three types of antiosteoporosis medications and HRT. New information regarding the risks of CVD among teriparatide users was also reported. The present study showed that users of denosumab, teriparatide, or bisphosphonate were significantly associated with lower odds of different cardiovascular events compared to those that did not use the specified medication and that the risks of CVD were the lowest in denosumab users, followed by teriparatide and then bisphosphonate; however, the use of HRT was associated with higher odds. Patients with osteoporosis with the following characteristics, women, older age, blue collar, or had DM, hypertension, or COPD, had higher risks of cardiovascular diseases, while those with hyperlipidemia, malignancy, or osteoporosis fracture had lower risks.

Our study showed that the use of bisphosphonate was associated with lower odds of CAD, PAD, HF, Af, and arrhythmia other than Af but higher odds of PE. These results were in agreement with previous studies describing bisphosphonate’s effect in reducing CVD mortality (Lyles et al., 2007; Rodríguez et al., 2020) but were in contrast with a meta-analysis demonstrating that bisphosphonate reduced arterial wall calcification but had no effect on arterial stiffness or on cardiovascular events (Kranenburg et al., 2016). However, the study population of this meta-analysis not only included patients with osteoporosis but also included patients with cancer, chronic kidney disease, and rheumatic disorders. In addition, there was no further stratifications for different cardiovascular events. In a population-based study, high cumulative doses of bisphosphonate significantly reduced both coronary and cerebrovascular events (Casula et al., 2020). The possible protective mechanism of bisphosphonate on CVD in our study may be related to it being a pyrophosphate analog. Pyrophosphate is a strong inhibitor of arterial calcification (Fleisch et al., 1965; Lomashvili et al., 2014). Bisphosphonates might, therefore, stimulate the inhibitory effects of pyrophosphate on arterial calcification (Bevi et al., 2005; Elmariah et al., 2010). Bisphosphonates have also been demonstrated to suppress macrophages (Mönkkönen et al., 1994) that oxidized LDL cholesterol to form atherogenic foam cells (Ross, 1999) and were, therefore, mentioned by previous studies that some types of bisphosphonates may intervene cholesterol biosynthesis (Ylitalo, 2000; Bevi et al., 2005). Due to such mechanisms, bisphosphonates might be able to help reduce the risk of and mortality from CVD or myocardial infarction (Lyles et al., 2007).

Although, in the present study, the decreased risk of Af was associated with the use of bisphosphonates, concerns exist about the risk of serious Af events associated with bisphosphonates from the previous literature (Black et al., 2007; Cummings et al., 2007), owing to its effect of altering intracellular ion concentration and pro-inflammatory, pro-fibrotic, and antiangiogenic properties (Howard et al., 2010; Pazianas et al., 2010). Some reviews found an increased risk of Af among bisphosphonate users (Loke et al., 2009; Bhuriya et al., 2010; Sharma et al., 2013; Sharma et al., 2014), whereas others did not (Karam et al., 2007; Mak et al., 2009; Kim et al., 2010; Lewiecki et al., 2010; Barrett-Connor et al., 2012). The mechanism supporting the antiarrhythmic effect of bisphosphonate was that antifibrotic effects had been demonstrated in human cells (Yang et al., 2013; Ye et al., 2015; Komatsu et al., 2016). As for the increased risk of PE among the bisphosphonate users in our study, a Denmark cohort showed that alendronate, clodronate, and etidronate (HR 1.37, 95% CI 1.23–1.51) were all associated with an increased risk of DVT/PE (Vestergaard et al., 2010). Older age (Tritschler and Aujesky, 2017) and immobilization after fracture (Pedersen et al., 2017) are also possible risk factors for PE. However, the absence of a dose–response relationship for etidronate with DVT/PE and an inverse relationship for alendronate may indicate a lack of a causal relationship between bisphosphonates and DVT/PE (Vestergaard et al., 2010). On the other hand, Lamberg et al. demonstrated a neutral effect of bisphosphonate on DVT/PE (Lamberg et al., 2010). Our study also showed that DVT had a decreased association with bisphosphonate. It was this study’s limitation that our sample size was not large enough to further analyze different effects of each subtype of bisphosphonate. The association between bisphosphonate and DVT/PE still calls for a large-scale RCT with a longer duration and DVT/PE as primary endpoints.

In the present study, the denosumab users had the lowest ORs for all the eight types of CVD, which might indicate a stronger protective effect against all CVDs than other medications. The protective relationship was consistent with a propensity score-matched cohort study of 5,046 patients comparing denosumab to alendronate users (Hsu et al., 2019). Among patients with a medication possession rate ≥60%, CVD was significantly lower for denosumab users than alendronate users (9.08% *vs.* 10.3%, respectively) (log-rank test, *p* = 0.0028) (Hsu et al., 2019). However, our findings were different from previous studies describing no impacts (Ferrieres et al., 2020; Lv et al., 2020) or a higher risk of CVD comparing denosumab to bisphosphonates in postmenopausal women (Seeto et al., 2021). Reasons for such discrepancy might be that different trials have different inclusion criteria for defining CVD and that not many trials used each specific cardiovascular event as the primary outcome. Since CVD events were not the primary endpoint of previous RCTs of antiosteoporosis medications, the total CVD events of these meta-analyses were few.

Two possible mechanisms may also help explain the effects of denosumab on the cardiovascular system. First, the long-term use of denosumab was shown to be effective in reversing or treating aortic arch calcification in patients undergoing hemodialysis (Suzuki et al., 2021). Second, denosumab had a similar mechanism as osteoprotegerin (OPG) on osteoclasts to treat osteoporosis. The OPG weakens osteoclast activity by blocking the interaction between the receptor activator of nuclear factor kappa B (RANK) and its ligand (RANKL). RANKL has been shown to be correlated with plaque destabilization and thrombosis based on its role in vascular calcium deposition and its prominent expression in advanced lesions (Collin-Osdoby, 2004; Sandberg et al., 2006). However, previous research has illustrated a deleterious effect of OPG on CVD by enhancing the adherence of leucocytes to the endothelial surface, the activation of the renin–angiotensin system (RAS), pro-inflammatory and pro-fibrotic effects, and the induction of endothelial dysfunction in the early stages of atherogenesis (Dutka et al., 2022). Whether denosumab has the same deteriorating effect as OPG on CVD or whether the administration of denosumab can suppress OPG still requires further explorations.

Another mechanism to explain the possible protective effect of denosumab on CVDs might be through the suppression of PTH. PTH may affect the cardiovascular system by inducing oxidative stress, necrotic cell death, cardiac hypertrophy (Schlüter et al., 1995), and vasodilatation (Schlüter et al., 1995). Although a high serum PTH level is not linked to coronary calcification (Arad et al., 1998), it has been shown to be an independent risk factor for cardiovascular mortality in patients with stable CAD (Pilz et al., 2010). It has been noted that PTH increased significantly from baseline after 1 month of starting denosumab and declined gradually but was still significantly greater than baseline at month 6 (Dempster et al., 2016). In contrast, Nakamura et al. demonstrated that PTH was significantly increased at 1 week but then gradually decreased to lower than baseline at month 4 of starting denosumab (Nakamura et al., 2015). The probable association of denosumab and the decreased risk of CVD may be similar to cinacalcet, a calcimimetic agent that activates the calcium-sensing receptor on the parathyroid tissue by lowering PTH (Chertow et al., 2012), and has been proved to have a protective effect on the cardiovascular system (Kawata et al., 2008; Ivanovski et al., 2009; Choi et al., 2012; Wu et al., 2014; Yu et al., 2017). Chertow et al. also showed that patients with secondary hyperparathyroidism had a neutral effect of cinacalcet on major cardiovascular events (Chertow et al., 2012). Taken together, whether the suppression of PTH by the long-term use of denosumab may be a relevant mechanism of reducing CVD also needs more research.

The present study revealed that the teriparatide use was associated with decreased risks of CAD, PAD, HF, Af, and arrhythmia other than Af, which was consistent with prior RCT (Cosman et al., 2020). The database of individual case safety reports (ICSRs) from 130 countries (1967–2020) also showed that teriparatide was associated with fewer CAD than alendronate (Rodríguez et al., 2023). However, this database also revealed that the teriparatide use was associated with Af, arrhythmias, and angina (Rodríguez et al., 2023). The limitation to this database was that patients with a history of CVD were not excluded. A possible mechanism may be that the PTH level was suppressed rapidly and persistently during the administration of teriparatide (Anastasilakis et al., 2008) and, therefore, decreased the risk of PTH-related CVD. Teriparatide may also reduce the risk of DVT by decreasing the rate of immobilization after fracture (Pedersen et al., 2017). Since teriparatide is usually prescribed to patients with severe osteoporosis at a high fracture risk, who may also have an elevated risk of CVD (Marcovitz et al., 2005; Tankó et al., 2005; Varma et al., 2008), the effect of teriparatide on CVD is worthy of future investigation.

Our study showed that the use of HRT was associated with higher odds of CAD, PAD, HF, arrhythmia other than Af, PE, and DVT. This finding is similar to many of the previous randomized or observational studies describing elevated risks of VTE but in contrast to the decreased risk of myocardial infarction among HRT users (Kim et al., 2020). One reason for such an inconsistency may be that HRT in our study comprised various kinds of formulations and regimens that might have different cardiovascular effects, but we did not have a sufficient sample size to evaluate different effects of each subtype of HRT. For instance, a previous meta-analysis of five RCTs (Komm et al., 2015) demonstrated that conjugated estrogens/bazedoxifene (CE/BZA) had an acceptable cardiovascular safety profile, with rates of stroke and CAD comparable to the placebo in healthy postmenopausal women. Another reason patients with HRT were associated with a higher risk of CAD in the present study may be that 77% of our study participants were aged 65 or older. A previous review showed that low-dose oral and transdermal HRT appears to be safe regarding the CVD risk in women within the first 10 years after menopause (Grodstein et al., 2006; Hodis et al., 2016), which may be ages 45–60. Initiating HRT at an older age may be an explanation for the elevated risk of CVD among our HRT users (El Khoudary et al., 2020).

DM and hypertension are well-known established risk factors for CVD, and patients with COPD may also have a higher risk of CVD due to smoking (Libby, 2002; Paulus and Tschöpe, 2013), which induces chronic systemic inflammation, rupture of atherosclerotic plaques, and might lead to the development of CAD and HF. The reason patients with hyperlipidemia had a lower risk of CVD may be related to a previous intervention with statins (Bhattarai et al., 2020; Mangione et al., 2022). Despite that, we only assessed denosumab (Prolia) in this study; denosumab (XGEVA) and bisphosphonate may also be used to treat cancer, and the lower association of CVD and malignancy may be due to the usage of denosumab or bisphosphonate. In addition, since antiosteoporosis medications were reimbursed if patients already had osteoporotic fracture, the lower risk of CVD in patients with an osteoporotic fracture may also be related to a higher usage of antiosteoporosis medications.

This study has the strengths of head-to-head comparisons of four kinds of antiosteoporosis medications, while previous studies usually compared only two kinds of antiosteoporosis medications. In addition, we provided more evidence of teriparatide on the lowered risk of CVD. The other is that we investigated the most comprehensive outcomes with more of CVD than previous studies, using a nation-wide, population-based dataset. However, our study has some limitations. First, the nested case–control and the observational design, rather than a RCT design, may have restricted our generalizability. Those with a diagnosis of osteoporosis but did not take antiosteoporosis medication may represent a particular group with specific characteristics for not taking antiosteoporosis medications, and such characteristics may be related to their CVD risks. Second, there were still un-measured confounders, such as diet, exercise, or smoking, that were not available in NHIRD and for which we could not control in the data analysis. Third, we did not provide evidence for those with longer antiosteoporosis medication uses since the numbers of patients with persistent use of the medicines were limited. Fourth, the relatively lower events of CVDs, PE, and DVT might have an association with the inconsistent findings in the follow-up and case–control analyses, which may influence the interpretation of our results. Fifth, whether bisphosphonate had been used in patients with cancer was not clarified. However, we excluded the individuals who had used antiosteoporosis medications before being diagnosed with osteoporosis.

## Conclusion

Denosumab, teriparatide, and bisphosphonate may have more protective effects against CVDs than HRT for patients with osteoporosis. Future large-scale head-to-head RCTs of use of antiosteoporosis medications, comparing antiosteoporosis medications to the placebo with different incident CVDs as primary outcomes, may provide more evidence. Physicians may need to consider the possible protective effects of different antiosteoporosis medications when choosing an adequate antiosteoporosis treatment for patients with osteoporosis.

## Data Availability

The original contributions presented in the study are included in the article/Supplementary Material. Further inquiries can be directed to the corresponding author.
