# Supplementary material for: Antiosteoporosis medications and cardiovascular disease: a population-based nationwide nested case–control study
Source: Front Pharmacol. 2023 Oct 10;14:1220174. doi: 10.3389/fphar.2023.1220174 (PMC10595014; doi:10.3389/fphar.2023.1220174)
Supplement: Supplementary file 1 [file DataSheet1.doc]

**
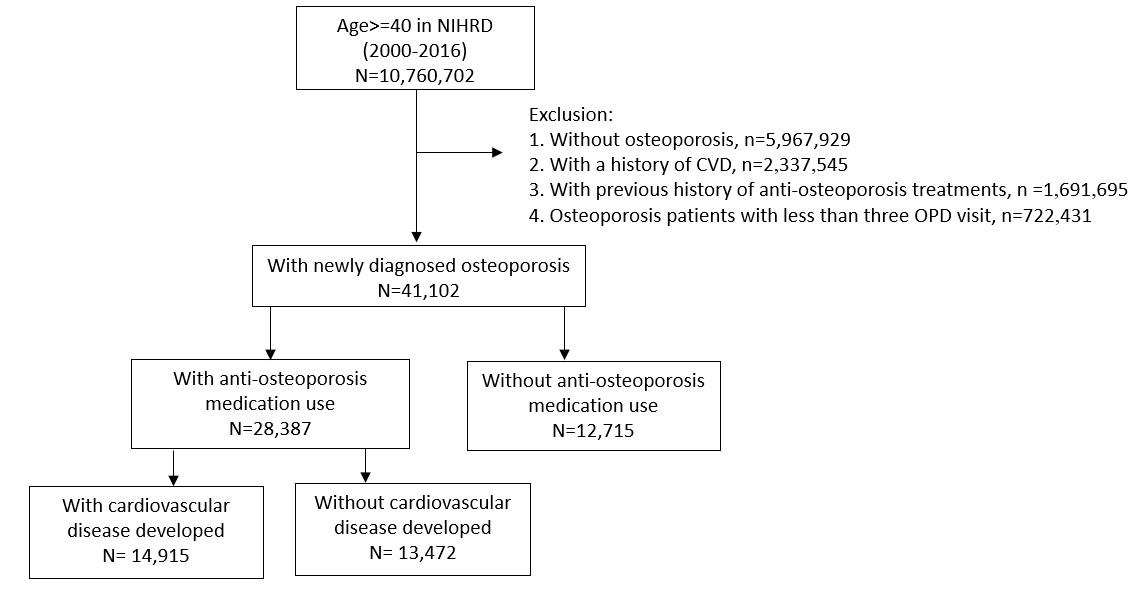
**

**Supplementary Figure 1.** Flow chart for identifying study cohorts with and without anti-osteoporosis medications, and case-control analysis groups

**Supplementary T**able 1

| **Coronary artery disease (CAD)** |
| --- |
| (ICD-10: I21.01~04, I21.09, I21.11, I21.19, I21.21, I21.29, I22.0~02, I22.08~09, I20.0, I24.0~01, I24.08~09, I25.10, I25.750~751, I25.758~759, I25.811, I25.110~111, I25.118~119, I25.760~761, I25.768~769, I25.710~711, I25.718~719, I25.812, I25.730~731, I25.738~739, I25.720~721, I25.728~729, I25.700~701, I25.708~709, I25.790~791, I25.798~799, I25.810, I25.812, Z95.1, Z95.5, Z98.61; ICD-9-CM: 410~412, 414.00~05, V45.81~82) |
| **Cerebrovascular disease** |
| (ICD-10: I66.01~03, I66.09, I66.11~13, I66.19, I66.21~23, I66.29~30, I63.30, I63.311~ 312, I63.319, I63.321~322, I63.329, I63.331~332, I63.339, I63.341~ 342, I63.349, I63.39, I63.6, I66.01~03, I66.09, I66.11~13, I66.19, I66.21~23, I66.29, I66.3, I66.9, I66.40, I66.411~412, I66.419, I66.421~422, I66.429, I66.431~432, I66.439, I66.441~442, I66.449, I66.49, I66.01~03, I66.09, I66.11~13, I66.19, I66.21~23, I66.29, I66.3, I66.8~9, I63.50, I63.511~512, I63.519, I63.521~522, I63.529, I63.531~532, I63.539, I63.541~542, I63.549, I63.59, I63.08~09, G45.0~02, G45.8, G46.0~02, G45.9, I67.841, I67.848, I67.89, I67.81~82, I67.89~90; ICD-9-CM: 362.34, 430~438) |
| **Heart failure (HF)** |
| (ICD-10: I11.0, I13.0, I13.2, I50; ICD-9-CM: 398.91, 402.01, 402.11, 402.91, 404.01, 404.03, 404.11, 404.13, 404.91, 404.93, 425.04~05, 425.07~09, 428) |
| **Peripheral artery disease (PAD)** |
| (ICD-10: I70.0~02, I70.20, I70.201~203, I70.208~209, I70.21, I70.211~213, I70.218~219, I70.220~223, I70.228~229, I70.230~235, I70.238~239, I70.240~245, I70.248~249, I70.25, I70.260~263, I70.268~269, I70.290~293, I70.298~303, I70.308~309, I70.310~313, I70.318~319, I70.320~323, I70.328~335, I70.338~ 345, I70.348~349, I70.35, I70.360~363, I70.368~369, I70.390~3, I70.398~403, I70.408~413, I70.418~ 422, I70.423, I70.428~ 435, I70.438~445, I70.448~449, I70.45, I70.460~463, I70.468~469, I70.49~493, I70.498~499, I70.50~503, I70.508~509, I70.51~513, I70.518~523, I70.528~535, I70.538~545, I70.548~550, I70.56~563, I70.568~569, I70.59~593, I70.598~603, I70.608~613, I70.618~623, I70.628~635, I70.638~645, I70.648~65, I70.66~663, I70.668~669, I70.69~693, I70.698~703, I70.708~713, I70.718~723, I70.728~735, I70.738~745, I70.748~750, I70.76~763, I70.768~769, I70.790~793, I70.798~800, I70.90~I70.92; ICD-9-CM: 250.7, 440.2~440.4, 443.81, 443.9, 785.4, 444.2, 444.8~444.9) |
| **Atrial fibrillation (Af)** |
| (ICD-10: I48.0~I48.2, I48.9; ICD-9-CM:427.31) |
| **Arrhythmia other than Af** |
| (ICD-10: I49.0~I49.02, I49.1~I49.4, I49.49~I49.5, I49.8~I49.9; ICD-9-CM: 427.9) |
| **Pulmonary embolism (PE)** |
| (ICD-10: I26.0, I26.09, I26.9, I26.93~94, I26.99; ICD-9-CM: 415.1) |
| **Deep vein thrombosis (DVT)** |
| (ICD-10: I82.400~403, I82.409~413, I82.419~423,  I82.429~433, I82.439~443, I82.449~453, I82.459~463, I82.469, I82.490~493,  I82.499, I82.4Y~4Y3, I82.4Y9, I82.4Z~4Z3, I82.4Z9, I82.50~503, I82.509~513, I82.519~523, I82.529~533, I82.539~543, I82.549, I82.550~553, I82.559~563, I82.569, I82.590~593, I82.599, I82.5Y~5Y3, I82.5Y9, I82.5Z~5Z3, I82.5Z9, I82.60~603, I82.609~613, I82.619~623, I82.629, I82.70~703, I82.709~713, I82.719~723, I82.729, I82.A, I82.A10~A13, I82.A19~A23, I82.A29, I82.B, I82.B10~B13, I82.B19~B23, I82.B29, I82.C, I82.C10~C13, I82.C19~C23, I82.C29, I82.80~813, I82.819, I82.890~891, I82.90~91; ICD-9-CM: 453.40~42, 453.50~52, 453.6, 453.70~77, 453.79~87, 453.89~90) |
| **Diabetes (DM)** |
| (ICD-10: E08, E09, E10, E11, E13; ICD-9-CM: 250) |
| **Hyperlipidemia** |
| (ICD-10: E78; ICD-9-CM: 272) |
| **Hypertension** |
| (ICD-10: I10~I13, I15~I16, I67.4; ICD-9-CM: 401~405, 437.2) |
| **Chronic kidney disease (CKD)** |
| (ICD-10: N18~N19; ICD-9-CM: 016.0, 095.4, 189, 223, 236.9, 250.4, 271.4, 274.1, 403~404, 440.1, 442.1, 446.21, 447.3, 572.4, 580-591, 593, 642.1, 646.2, 753, 984) |
| **Chronic obstructive pulmonary disease (COPD)** |
| (ICD-10: J44.0~1, J44.9, J45.20~J45.22, J45.30~32, J45.40~42, J45.50~52; ICD-9-CM: 490-496) |
| **Obesity** |
| (ICD-10: E66; ICD-9-CM: 278) |
| **Malignancy** |
| (ICD-10: C00-C14, C15~C26, C30~C80, except C42 or C59, C60~C80, C7A, C7B, C81~C96, D00~D09, D37~D48; ICD-9-CM: 140~149, 150~159, 160~165, 170~176, 179~209, 230~239) |
| **Liver cirrhosis** |
| (ICD-10: K74; ICD-9-CM: 571) |
| **End-stage renal disease (ESRD)** |
| (ICD-10: N18.6; ICD-9-CM: 250.4, 403~404, 580~588, 593, 791.0, V13.03, V42.0, V45.1, V56) |
